# On the tear resistance of skin

**DOI:** 10.1038/ncomms7649

**Published:** 2015-03-27

**Authors:** Wen Yang, Vincent R. Sherman, Bernd Gludovatz, Eric Schaible, Polite Stewart, Robert O. Ritchie, Marc A. Meyers

**Affiliations:** 1 Materials Science and Engineering Program, University of California, San Diego, California 92093, USA; 2 Materials Sciences Division, Lawrence Berkeley National Laboratory, Berkeley, California 94720, USA; 3 Advanced Light Source, Lawrence Berkeley National Laboratory, Berkeley, California 94720, USA; 4 Department of Materials Science and Engineering, University of California, Berkeley, California 94720, USA; 5 Department of Mechanical and Aerospace Engineering, University of California, San Diego, California 92093, USA; 6 Department of NanoEngineering, University of California, San Diego, California 92093, USA

## Abstract

Tear resistance is of vital importance in the various functions of skin, especially protection from predatorial attack. Here, we mechanistically quantify the extreme tear resistance of skin and identify the underlying structural features, which lead to its sophisticated failure mechanisms. We explain why it is virtually impossible to propagate a tear in rabbit skin, chosen as a model material for the dermis of vertebrates. We express the deformation in terms of four mechanisms of collagen fibril activity in skin under tensile loading that virtually eliminate the possibility of tearing in pre-notched samples: fibril straightening, fibril reorientation towards the tensile direction, elastic stretching and interfibrillar sliding, all of which contribute to the redistribution of the stresses at the notch tip.

## Abstract (web summary)

It is known that skin has a large tear resistance, but little is known of the mechanism behind this. Here, the authors carry out a structural analysis of rabbit skin to show how the deformation of collagen fibrils in the skin results in a strong resistance to tear propagation.

## Graphical abstract

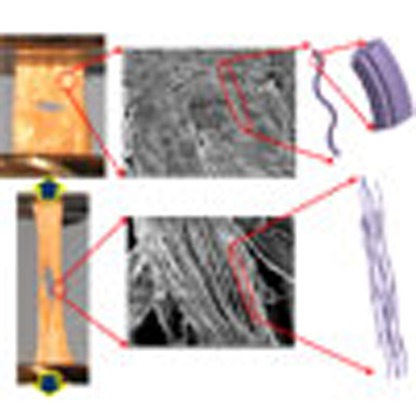

Vertebrates are covered with organ skin, which provides protection from the environment, temperature regulation, camouflage, thermal energy collection and a host for embedded sensors^1^. Skin consists of three layers, epidermis, dermis and endodermis, with mechanical properties dictated primarily by the dermis, the thickest layer. Its major constituents are type-1 collagen and elastin: collagen provides mechanical resistance to extension, whereas elastin accommodates the deformation^2^. To fulfill its multifunctional role, skin must possess a tailored mechanical response to accommodate the body’s flexibility and movement coupled with damage minimization strategies to prevent tearing.

Research into skin’s mechanical properties began in 1831 when Guillaume Dupuytren^3^ observed a patient who had stabbed himself over the heart three times with a stiletto having a circular cross-section. Doubting the patient’s truthfulness due to the elliptical shape of the wounds, Dupuytren found that perforations made from an awl may either narrow or broaden depending on the tension of the skin across the wound. This led to Langer’s proposal of lines representing the anisotropic nature of the skin that follow directions where the skin is under most tension^4^. The existence of Langer’s lines is well recognized; indeed, surgeons find that incisions made along the lines close easily and heal rapidly, whereas incisions perpendicular to the lines tend to pull open, with prolonged healing and scarring^5^.

Skin is often considered as a nonlinear-elastic material with low strain-rate sensitivity^6^,^7^. Most work on its mechanical deformation has focused on the collagen, the main structural component of the dermis^8^. Deformation in collagen involves several distinct stages^9^. In arterial walls, for example, collagen fibril straightening, reorientation and elastic stretching have all been identified^10^,^11^,^12^, akin to their alignment on stretching in tendons^13^,^14^. Constitutive models are based on phenomenological curve-fitting^15^, energy-based formulations^6^ and physically based relationships^16^,^17^,^18^.

Far less is known about the tearing of skin. Fracture-energy values have been measured for rhinoceros^19^ and rat^20^ skin, and the adherence of skin grafts estimated during peeling from a wound surface^21^. Skin appears to have superior tear resistance to natural materials, for example, hevea plantations^22^,^23^ and wheat gluten^24^, which are used in synthetic materials specifically to provide tear resistance. However, although the importance of tear strength and its dependence on fracture energy has been noted^25^, there are few, if any, studies that directly relate tear resistance to the salient micro-mechanisms of deformation and fracture in the skin.

This study addresses skin’s damage minimization strategies to prevent tearing. We attribute skin’s tear resistance to the nano/micro-scale behaviour of the collagen fibrils using mechanical and structural characterization involving *in situ* tension loading with small-angle X-ray scattering (SAXS) and scanning electron microscopy (SEM), together with ultrahigh-resolution SEM and transmission electron microscopy (TEM). As ~60% of skin is collagen^15^, which is primarily responsible for its mechanical properties, the role of elastin is not considered as it is only relevant at low strains^26^,^27^. Consequently, we validate our measurements using a constitutive equation derived from a physical analogue of skin’s collagen fibrils, comprising steel wires shaped into a configuration that can be analysed analytically. The tear resistance is due to the synergistic activation of four principal deformation mechanisms, which we identify and quantify the straightening and stretching of collagen fibrils, reorientation of fibrils towards the force application direction, and the sliding of fibrils by the deformation and reformation of bonds between them.

## Results

### Tensile response of skin

**Figure 1:**
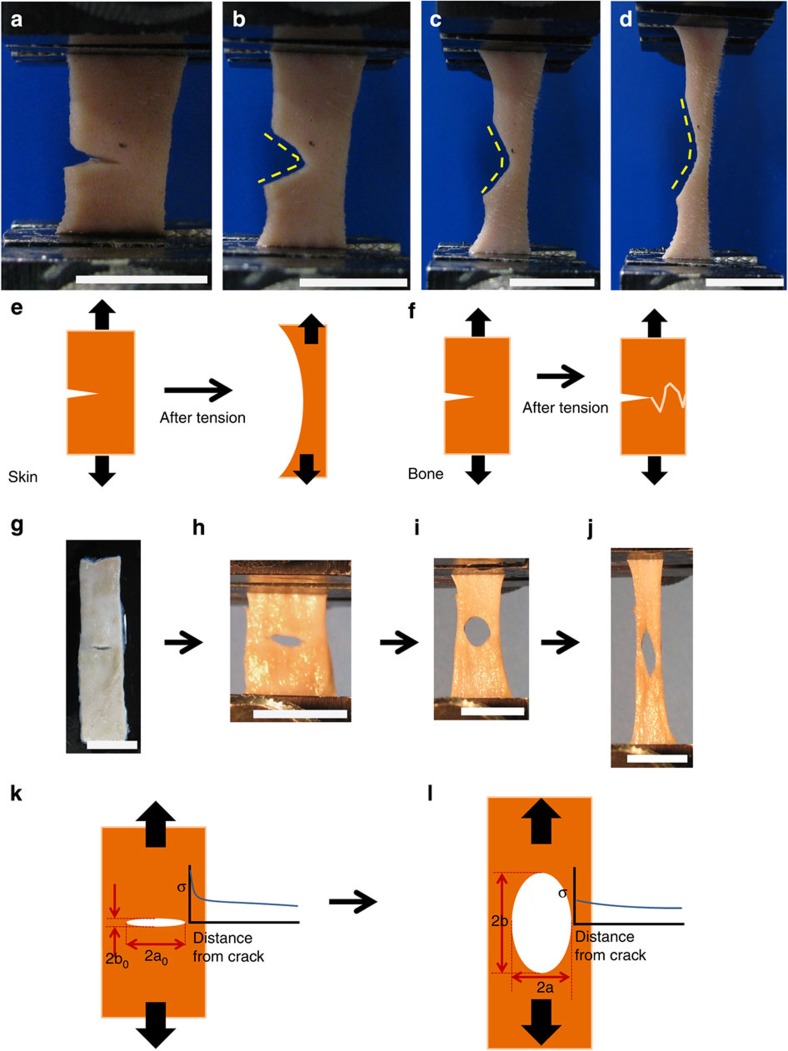
Tear resistance of skin in comparison to bone materials. (**a**–**d**) The sequence of events where rabbit skin, containing an edge notch or tear (of a length half the lateral specimen dimension), is strained under uniaxial tensile loading; the notch does not propagate but progressively yawns open under tensile loading. (**e**) Schematic illustration of skin with a pre-crack under loading; the crack does not propagate but instead blunts. (**f**) Corresponding schematic of bone (transverse orientation) with a notch under loading; the crack (white line) often propagates in a zig-zag pattern with multiple crack deflections. (**g**–**j**) The deformation of a central notch in skin loaded in tension. Distortion of a central notch as specimen of rabbit skin is extended uniaxially. There is no increase in the initial length of the cut. (**k**,**l**) The notch root radius increases with axial extension of the specimen, with a consequent decrease in stress concentration. This is enabled by local straightening and stretching of fibres and by interfibrillar sliding. Scale bar in (**a**–**d**), (**g**–**j**) is 10 mm.

We first established the tensile stress-strain response of skin with hydrated edge-notched specimens to demonstrate its dramatic resistance to tearing (Fig. 1a–d), which we relate to synergistic structural changes occurring in the dermis during straining. Our experiments show that a notch in the skin did not propagate or induce fracture, it simply opened and blunted (Fig. 1e). This response is distinct from that of bone and tooth dentin, which are also collagenous materials but with mineral crystals^28^,^29^,^30^, where a notch can initiate cracking and failure (Fig. 1f), and from natural rubber, where again a small cut can readily cause fracture. This experiment pertains to the opening of a tear at the edge of the skin. These experiments are done on hydrated specimens to reflect reality. However, the mechanical response is significantly altered by decreasing the water content, as described in the Supplementary Discussion. The corresponding behaviour of an internal tear (Fig. 1g–j), which is more likely encountered in surgery, illustrates how an initially straight cut gradually deforms along a trajectory idealized by an ellipse that decreases its major axis (2*a*) and increases its minor axis (2*b*) until inversion occurs, as has been demonstrated computationally at the nanometre scale^31^,^32^. This change in notch geometry, shown in Fig. 1k,l, acts to diminish the stress concentration at the tip, as the local stress at the notch tip, *σ*_tip_, is related to the globally applied stress, *σ*_app_, by *σ*_tip_=*σ*_app_ (1+2*a/b*). When the minor axis is zero, the local stress is infinite; as the minor axis 2*b* increases and the major axis 2*a* decreases, this stress decreases. We show how this extraordinary flaw tolerance of skin is related to the reorganization of the collagen at any region of stress concentration.

**Figure 2:**
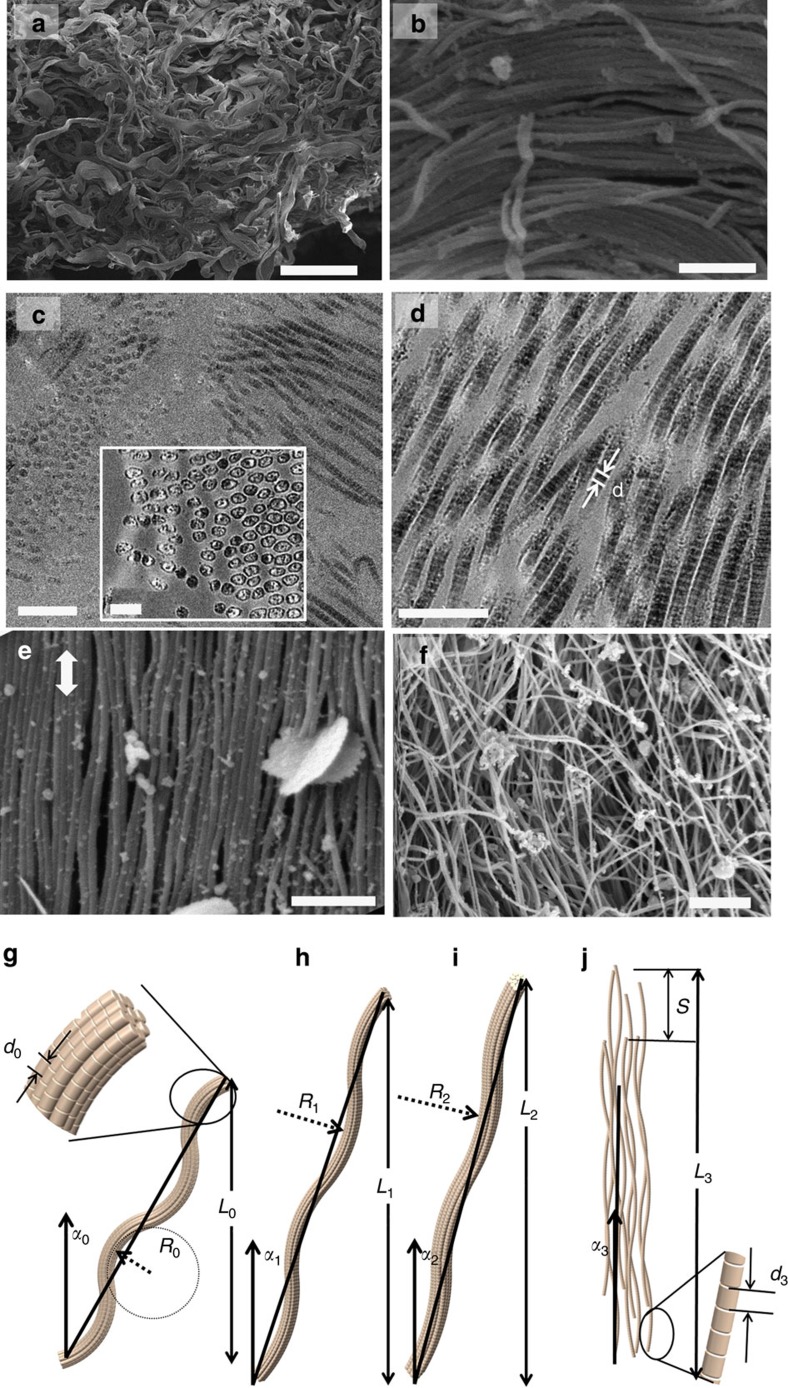
Evolution of fibril and fibre configuration during tensile extension. (**a**) Disordered arrangement of curved collagen fibres (SEM). (**b**) High magnification of **a**, collagen fibrils (~50 nm diameter) comprising each fibre (~1–10 μm diameter; SEM). (**c**,**d**) Collagen fibrils in section plane parallel to skin surface including detail of sectioned fibrils (inset in **c**) and wavy structure (TEM). (**e**) Collagen fibrils at notched side are delaminated, aligning close to the tension direction after loading. The loading direction is shown by the arrow, (**f**) collagen fibrils at unnotched side are delaminated/relaxed after loading/unloading. (**g**–**j**) Schematic of mechanisms of fibril deformation and failure under tension: (**g**) original configuration; (**h**,**i**) straightening and reorientation of fibres with projected length in tensile direction increasing from *L*_*0*_ to *L*_*1*_, and *L*_*2*_ (**j**) separation into fibrils; elastic stretching through the increase in collagen *d* spacing from *d*_*0*_ to *d*_*3*_, and sliding (schematically shown by *S*), increasing length in tensile direction to *L*_*3*_. *R*_0_−*R*_2_ are the radii of curvature of collagen during stretching. Scale bars in **a**–**f** and the picture inset in **c** are 50 μm, 500 nm, 500 nm, 500 nm, 1 μm, 2 μm and 200 nm, respectively.

Before testing, the collagen fibres show a disordered, curvy morphology (Fig. 2a). Each fibre has a diameter of 5–10 μm and contains hundreds of ~50-nm diameter collagen fibrils (Fig. 2b). TEM of the collagen fibrils reveals their principal orientations: nearly parallel and nearly perpendicular to the plane of the foil (Fig. 2c), with a curved trajectory; their *d*-spacing, measured at 55 nm (Fig. 2d), is lower than the actual value because the fibrils are inclined to the plane of observation. Under load, fibre straightening and reorientation occurs towards the direction of straining, as illustrated in Fig. 2g–j. After loading, the collagen fibrils are aligned parallel, straightened and separated on the notched side, but relaxed from straightening and delamination on the unnotched side (Fig. 2e,f). SEM images demonstrate that the collagen fibres straighten and reorient leading to their separation into fibrils from the action of the interfibrillar shear and tensile stresses (shown later).

### Mechanisms of deformation

The sequence of events can be analysed in terms of four mechanisms (Fig. 2g–j). One fibre with a reduced number of fibrils is used to schematically represent the process of deformation (Fig. 2g). The fibre stretches and reorients itself, increasing its projected length in the tensile direction from *L*_0_ to *L*_1_ and *L*_2_ (Fig. 2g–i). This takes place by increasing the radius of curvature of the initially curved fibres from *R*_0_ to *R*_1_ and *R*_2_; due to stretching, the angle with the tensile axis decreases from *α*_0_ to *α*_1_, and *α*_2_ (Fig. 2g–i). As the fibres are straightened, shear strains develop between the fibrils because of kinematic requirements. At a critical juncture, the shear stresses at the interfaces exceed the interfacial cohesive strength and the separation of fibrils ensues, leading to the last stage of deformation in which extensive interfibrillar displacement occurs (Fig. 2j). The displacement between two adjacent fibrils is indicated as *S* and the length along the tensile direction is now *L*_3_ (Fig. 2j). By the end of deformation, the *d*-spacing of collagen has increased from *d*_*0*_ to *d*_*3*_, as shown in Fig. 2g–j. Separation of the fibres into fibrils is shown in Fig. 2j.

**Figure 3:**
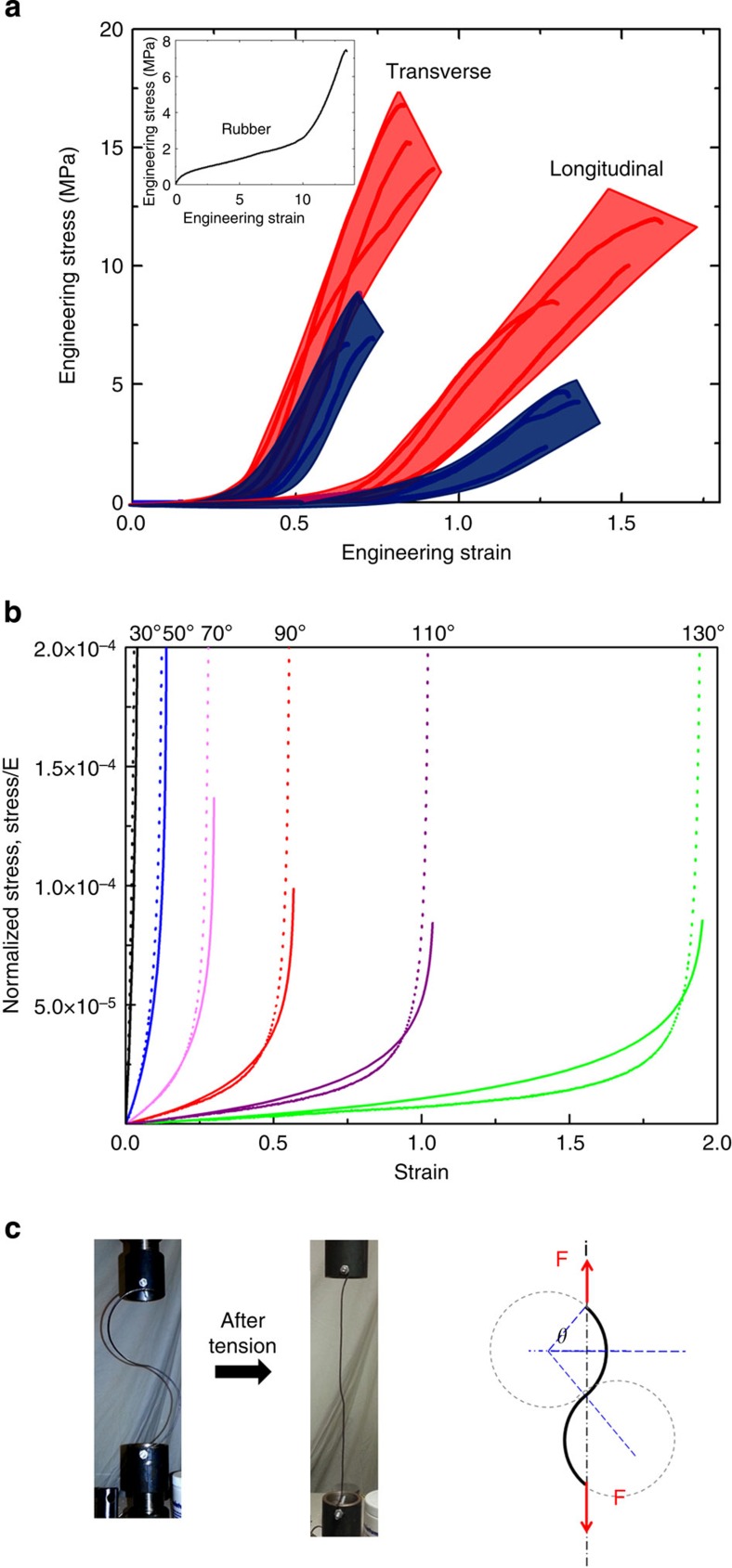
Experimental and predicted tensile response of a wavy structure simulating collagen in skin. (**a**) Stress-strain curves of rabbit skin in longitudinal (parallel to backbone, perpendicular to Langer’s lines) and transverse (perpendicular to backbone) orientations, at strain rates of 10^−1^ (red band) and 10^−3^ s^−1^ (blue band). Skin displays higher strength at higher strain rates. Inset shows tensile response of latex, with much higher tensile strains determined by the degree of vulcanization. (**b**) Modelling of stress-strain curves of skin with Castigliano's therom (dashed lines) and by experiments using steel wire, composed of segments of circles (full lines). (**c**) Steel wire before and after stretching. The wire curvature (shown in schematic drawing) is defined by the central angle *θ*_0_ (~30° to 130°), which determines the maximum strain. Experimental and mathematical predictions indicate good agreement reflecting the characteristic response of skin.

Figure 3a shows the stress-strain curves of unnotched rabbit skin at two different strain rates, differing by a factor of 100: 10^−1^ and 10^−3^ s^−1^. The plots represent a number of experiments (up to eight tests conducted for each condition) and the bands reflect the variation among individual results. The principal effect of increasing the strain rate is to increase the maximum stress, consistent with previous findings^6^,^7^, which we relate to the viscous effects of the extracellular matrix, including the sliding of collagen fibrils. Two orientations were tested: parallel and perpendicular to the backbone of the rabbit, which are, respectively, perpendicular to and along the Langer’s lines^7^. The maximum strains are lowest along Langer’s lines, as expected. The tensile curves show three regions, characteristic of many collagenous materials^13^,^33^: I–toe, II–heel and III–linear region. For comparison, the tensile response of isotropic, latex rubber is plotted in the inset of Fig. 3a; this has a characteristic shape with an inflection point followed by a steep slope increase associated with entropic effects. In the dermis, collagen does not display this behaviour; indeed, there are significant differences between the plots of the two materials. In the skin, the slope increases monotonically with increasing strain, until the linear region is reached. The skin shows higher strength (~15 MPa) at the strain rate of 10^−1^ s^−1^, than at the strain rate of 10^−3^ s^−1^ (~8 MPa), the maximum stress decreasing from the prominence of interfibrillar sliding at low strain rates. Polymeric chains in rubber, conversely, are connected by strong bonds (for example, vulcanization) such that stretching of the structure is dictated by other mechanisms. In collagen, higher strain rates leave less time for interfibrillar sliding and owing to increased viscous forces, the fibres can carry more stress. These results are consistent with human skin tested parallel and perpendicular to Langer’s lines^34^,^35^,^36^; the strength was also higher (~17–28 MPa) and the maximum strain lower (~0.5–0.6) parallel to the Langer's lines, compared with the corresponding strength (~10–16 MPa) and strain (~0.4) perpendicular to the lines.

### Constitutive response and modelling

We modelled the tensile stress-strain response of skin by using a steel wire composed of circular segments. This new model is superior to the use of a sine-function^18^,^27^, zig-zag^37^ or a helical shape^16^,^17^ because opposite segments are always continuous, independent of the radius; moreover, it enables analytical solutions to be derived. Sections of semicircles were connected consecutively, a geometry which is pertinent as there are no discontinuities in slope; this form accurately represents the *in vivo* arrangement of collagen. This is preferable to previous approaches because of its ability to control the maximum attained strain while maintaining an accurate representation of the skin. Supplementary Fig. 1 further justifies the selection of the chosen shape. Figure 3c shows one example of the collagen shape. The maximum strain is determined by the angle *θ* that defines the circular segments, increasing with rising *θ*. For instance, the maximum strain corresponding to a total rectification of the segments at an angle *θ*=90° is equal to 0.57. *θ* is the central angle of one quarter of the model; circular segments with central angles of 30°, 50°, 70°, 90°, 110° and 130° for a radius *r* of 120 mm were used to model the shapes of the collagen. Fig. 3c shows the metal wire in the initial and fully stretched configurations. We used Castigliano’s theorem^38^ to derive the stress, *σ*_0_ (normalized by the Young’s modulus, *E*), which we compare with the experimental results from steel wires, shown by the solid lines in Fig. 3b. Specifically, the extension of the steel spring was analysed assuming a purely elastic response of circular beam segments in tension:


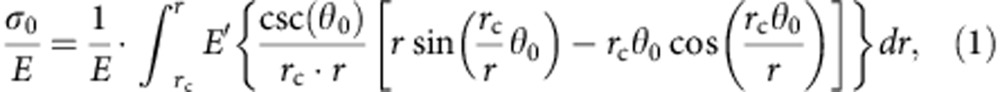


where *E′* is a pseudo-modulus (determined from the geometric shape of the wire), *θ*_0_ is the initial central angle of the 1/4 circular segments (Fig. 3b) and *r*_c_ is the initial circle radius. The strain increment, *dε*, can be obtained directly from the change in radius *r* as the segment is stretched:


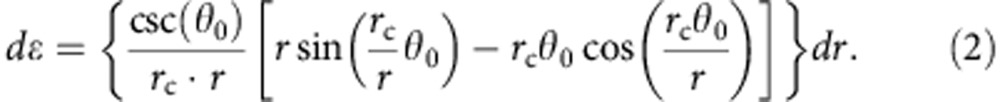


The dashed lines in Fig. 3b show the model predictions from equations 1 and 2.

The time-dependent component can be expressed by the Maxwell model, with the elastic spring (equations 1 and 2) and a dashpot in series. The viscous contribution is due to hydrogen bonding between the fibrils, which, on being disrupted and reformed, allows their time-dependent sliding.

To include the non-elastic terms from interfibrillar sliding, we assume a simple spring/non-linear dashpot series model where the total strain *ε*_t_ is given as the sum of the elastic *ε*_el_ and viscous *ε*_η_ strains: *ε*_t_=*ε*_el+_*ε*_η_. The viscous term can be represented by a simple Newtonian response: 
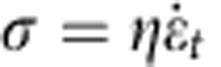
, where *η* is the Newtonian viscosity, such that the viscous strain is given by:


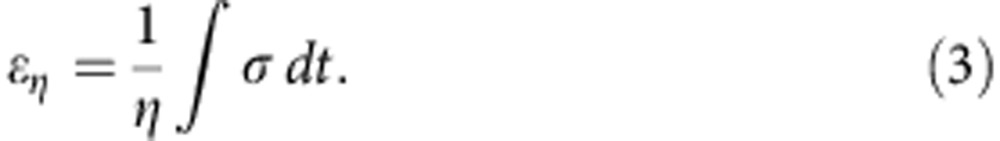


**Figure 4:**
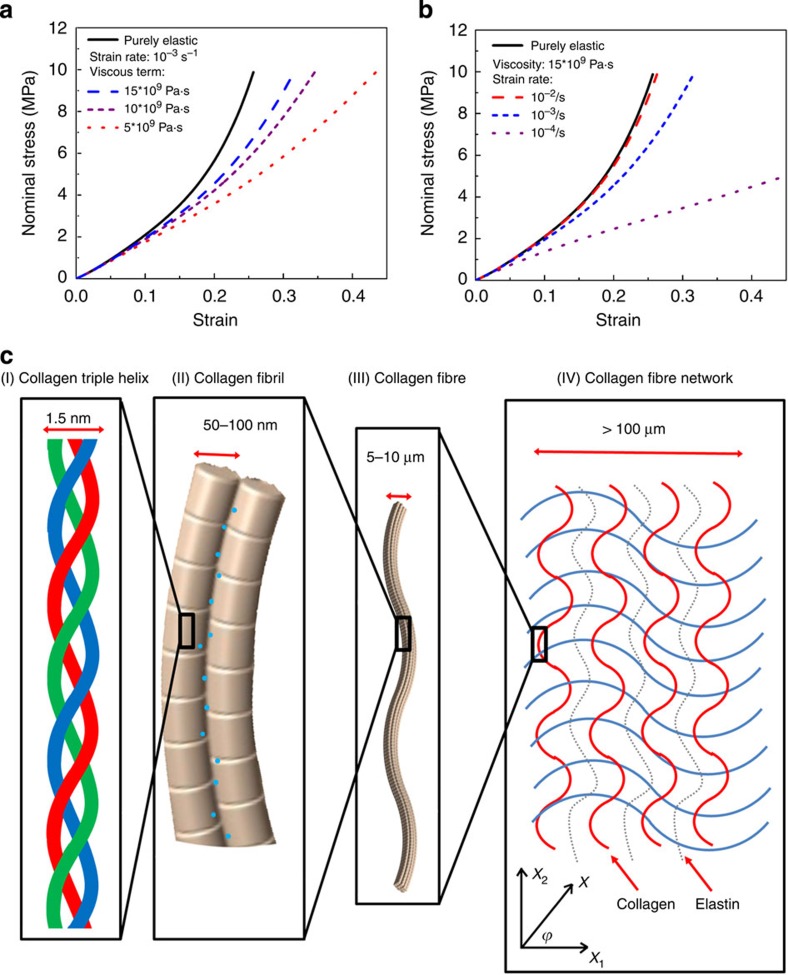
Viscosity and hierarchical structure. (**a**) Effect of viscosity on the stress-strain response of a non-linear elastic material. (**b**) Effect of strain rate, at a constant viscosity, purely elastic response at 10^−3^ s^−1^. (**c**) Actual skin has a hierarchical structure spanning the nanoscale of twisted peptide chains to the microscale of wavy collagen and elastin fibres. The proposed wire model only addresses structure at the ~50 nm to 10 μm dimensions, as depicted by levels II and III in the schematic. Blue dots in II represent hydrogen bonds and water molecules.

It is simpler to use a polynomial fit to the elastic constitutive equation of the form 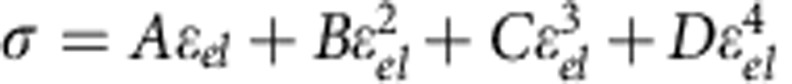, where *A*, *B*, *C* and *D* are fitting constants, leading to:


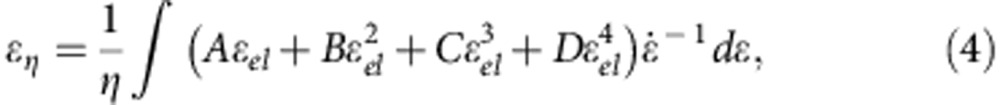


where 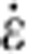 is the strain rate. We should emphasize that the viscous component comes from the breaking of interfibrillar bonds, which results in sliding between them. Thus, the fractional area where viscous flow takes place is a small number; as such, the viscosity used in equation 4 is an ‘effective’ viscosity. The resulting stress-strain response of the wire is modified as a function of viscosity (at a constant strain rate) in Fig. 4a, and strain rate (at a constant viscosity) in Fig. 4b. These calculations show in schematic manner how the viscosity influences the mechanical response. As the samples dry, the viscosity increases and the overall response is altered. This is predicted by the modelling of Gautieri *et al*.^39^, as shown in the Supplementary Discussion.

The wire model is a simple representation of almost two levels of the hierarchy of the skin. Figure 4c shows four levels of such hierarchy (considering primarily collagen), specifically: I (sub-nanometre) level—collagen molecule, II (nanometre) level—collagen fibrils, III (micrometre) level—collagen fibres, arranged in a ‘curvy’ geometry, and IV (mesoscale) level—collagen fibres with two orientations creating a fabric with orthotropic response. More complex models can be developed^40^ but for the purposes of this analysis the one presented in Fig. 4c suffices. The model focuses on levels II and III. Translating this to the mesoscale in level IV, and incorporating anisotropy, can provide the orientation-dependent mechanical response between the orthogonal axes *ϕ*=0 (direction of the Langer's lines) to 90° (perpendicular direction) in terms of the strains in directions *x*_1_ and *x*_2_ by:


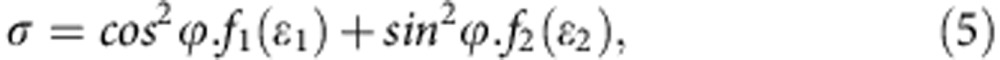


where *f*_1_ and *f*_2_ are different functional dependencies of the stress. This leads to predictions of the stress-strain response as a function of the orientation in the skin, as described in the Supplementary Discussion section, specifically in Supplementary Fig. 2, which captures the essential features of the experimental data in Fig. 3a.

### Synchrotron X-ray characterization

**Figure 5:**
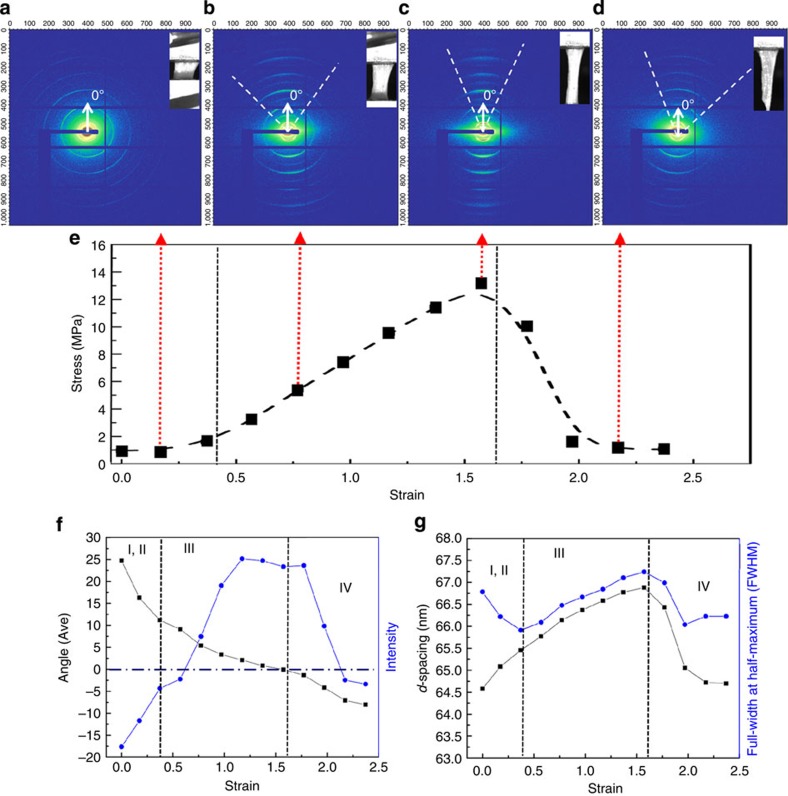
SAXS analysis of skin in tension. Variation in SAXS peak-intensity, orientation angle, collagen fibril *d*-spacing and full-width-at-half-maximum (FWHM), from tensile tests on rabbit skin. (**a**–**d**) Diffraction patterns: arcs show orientations of fibrils, images of the sample shown at top-right corners, (**a**) collagen fibrils randomly oriented to tensile axis, shown by constant intensity of diffraction pattern circles, (**b**) fibrils become gradually aligned in tension direction, (**c**) fibrils aligned along tensile axis, (**d**) fibrils fractured and relaxed. (**e**) During tensile test, 13 stress-strain data points (black dots) were recorded at 5 s intervals; four stages were identified. (**f**) Angle of normal to the tensile axis (black dots) versus intensity of fibrils (blue dots) as a function of strain, and (**g**) *d*-spacing (black dots) and FWHM (blue dots) of fibrils as a function of strain. Four stages: *I-toe* and *II-heel*, curved collagen fibrils straighten, rotate, stretch (*d*-spacing increases), *III-linear*, fibrils continue to rotate and stretch, orienting completely along tensile axis (angle=0°), but also slide and delaminate; *IV-fracture*, fibrils fracture and curl back (angle deviates from 0°, *d*-spacing, FWHM and intensity decrease).

**Figure 6:**
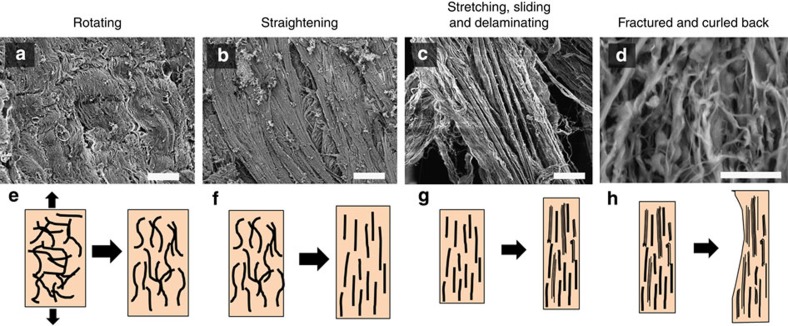
Mechanistic stages of the tensile loading of skin. SEM images (**a**–**d**) and schematic drawings (**e**–**h**) of the mechanisms during the four stages of tensile loading of rabbit skin, black arrows in **a** and **e** represent the direction of tension testing. (**a**,**e**) Curved collagen fibrils are oriented along the tensile axis; (**b**,**f**) collagen fibrils are straightening, larger and larger amount of the fibrils re-orient close to the tensile axis; (**c**,**g**) collagen fibrils are stretching, sliding, delaminating and orientated completely along the tensile axis; (**d**,**h**) collagen fibrils are fractured and curled back. Scale bars in **a**–**d** are 20, 20, 20, 50 μm, respectively.

We used *in situ* SAXS^41^ with a synchrotron X-ray source to investigate this reorganization of the collagen fibrils in the skin during tensile loading, combining these data with *in situ* structural observations of the collagen behaviour in the environmental SEM under stretching. SAXS has been used previously to study collagen^18^,^42^,^43^, specifically the uniaxial and biaxial directional stretch of bovine pericardium and the collagen structure at different temperatures and degrees of hydration. Here, we determined a stress-strain curve for skin exhibiting the three characteristic toe-, heel-, and linear-shaped regions^8^,^13^,^44^ (stages I–III) with a stage IV representing failure (Fig. 5a–e). The first three stages display a characteristic J-shape, which has been seen for collagen in other organs^8^,^11^,^12^,^13^. Each point on the curve represents a SAXS measurement during tensile loading with 13 points exposed to X-rays. The four data points at the ends of the red dashed line arrows in Fig. 5e are used to discuss the structural changes shown in Fig. 5a–d. In the diffraction patterns of the four points (Fig. 5a–d), the arcs represent the distributions of orientation of the collagen fibrils; the radii of the arcs indicate their *d*-spacing evolution. Figure 5f,g show, respectively, the evolution of the central angle of orientation of the collagen fibrils and their *d*-spacing. Evaluation of the results in Fig. 5, combined with *in situ* SEM observations (Fig. 6), permits the identification of the four salient mechanisms underlying the tear resistance of skin during tensile straining—in four stages marked I–IV in Fig. 5f,g.

### Stage I and II (toe and heel)

The skin was moderately stretched before loading because of the gravity acting on the wet samples. No clear mechanistic distinction was observed in stages I and II, since due to the dose limit, only three data points were obtained. The diffraction pattern in Fig. 5a (at the beginning of tensile testing) displays almost a continuous circle, suggesting that the collagen fibrils are arranged at widely varying angles. During these stages, the collagen fibrils straighten (Fig. 6b,f) and rotate towards the tension axis (Figs 5f and 6a,e). The fibrils also stretch, as the collagen *d*-spacing increases (Fig. 5g). Despite the increasing strain, the toe and heel stages show little increase in stress, consistent with the wire model data, which suggest that during this period more strain is taken up by straightening than by stretching.

### Stage III (linear)

The collagen fibrils continue to rotate, as *α* (fibril angle with the tension axis) drops from ~10 to ~0°. The fibrils also become more uniformly aligned, with Herman’s orientation factor increasing from 0.24 to 0.76 (data not shown). Herman’s orientation factor defined as ½(3cos^2^Φ−1), where Φ is the angle between the orienting entity and fibre axis, quantifies orientation on a scale from 0 (random distribution) to 1 (perfectly oriented/aligned). This is seen visually as the SAXS peak transforms from a circle to an oriented arc (Fig. 5a–c). This peak correspondingly grows in intensity (Fig. 5f), which also reflects the recruitment of greater numbers of fibrils into common alignment. The realignment of the collagen fibrils possibly increases the modulus locally, which would elevate local stress and precipitate failure at this stage. Simultaneously, the *d*-spacing of collagen fibrils increases from 64.5 to 66.9 nm, indicating that the collagen is still extending elastically. However, this small elastic strain of ~0.037 is not sufficient to accommodate the applied strain, which can be as high as 0.5 in this stage. Hence, the mechanisms of inter- and intrafibrillar sliding become major contributors to accommodate the imposed strain. Delamination of collagen fibrils is observed (Fig. 6c), consistent with the SAXS peak becoming broader (full-width-at-half-maximum (FWHM) increases, Fig. 5g), owing to the defects introduced into the previously well-ordered fibrils. In this stage, the main mechanisms are reorientation, stretching, sliding, and delamination of collagen fibrils (Fig. 6e,g).

### Stage IV (fracture)

In stage IV, the collagen fibrils fracture and curl back upon unloading (Fig. 6d). The fibrils return to a wider range of orientations, so that the SAXS peak concomitantly decreases in intensity, and the central angle of orientation drifts away from the axis of tension. Owing to unloading, the collagen *d*-spacing (Fig. 5g) and the FWHM of the SAXS peak both decrease, as the fractured collagen returns to a shorter and more well-ordered *d*-spacing.

Thus, multiple mechanisms operate in the collagen under tensile loading to provide skin with its extraordinary tear resistance: rotation, straightening, stretching, sliding and delamination. The first three mechanisms provide the strain to induce large shape changes within the elastic regime; these mechanisms also permit the re-alignment of collagen around any tear in the skin to ensure its blunting.

In conclusion, we have shown the remarkable tear resistance of skin to be associated with specific mechanisms within the collagen. This behaviour, especially the ability of collagen fibrils to slide past each other, contrasts with natural rubber vulcanizate, where ‘nicked’ specimens will readily tear at low loads^45^. Clearly, the role of collagen fibrils varies significantly in biological materials. In bone^29^,^46^, sliding between the collagen fibrils forms the basis of ‘plasticity’ and provides a bilinear uniaxial stress-strain response; indeed, collagen fibrils interact with cracks contributing to toughness. In certain fish scales^47^,^48^, the Bouligand-type structure, with collagen fibrils oriented in different directions, acts as a tough foundation to the highly mineralized surface to provide resistance to both penetration and fracture. In such biomaterials, the collagen fibrils are mineralized and initially straight. In contrast, the collagen fibrils in the skin are initially curvy and highly disordered. We have shown how these curvy collagen fibrils act to enhance skin’s tear resistance through their rearrangement towards the tensile-loading direction, with rotation, straightening, stretching, and sliding/delamination before fracture. The rotation mechanisms recruit collagen fibrils into alignment with the tension axis at which they are maximally strong or accommodate shape change (for example, blunting a tear); straightening allows strain uptake without much stress increase, sliding allows more energy dissipation during inelastic deformation. Such reorganization and sliding of the fibrils are responsible for stress redistribution (blunting) at the tips of tears and notches. It is the synergy of these four mechanisms that confers the extraordinary resistance to tearing in skin, which in itself is a requisite for the survival of organisms.

## Methods

### Materials

Sexually mature female New Zealand white rabbits (*Oryctolagus cuniculus*) were obtained from a breeder in Lake Elsinore, California (Da Le Ranch), USA. The animals were purchased and delivered as commercially available dead rabbits, in order to avoid the ethical implications of working with live animals. The hair was shaved carefully on the animal without damaging the skin before testing. The skin was pulled from the rabbit body with a minimum of cuts. Skin samples along both the transverse and longitudinal directions, with dimensions of 10–15 mm in width and 25 mm in length, were taken from the sides and back of the rabbit. The epidermis was not removed as it was presumed that the mechanical properties of the skin would not be affected by the very thin epidermis layer. Skin samples that were not tested immediately were stored in the frozen state, as prescribed by Marangoni *et al*.^49^

In total, three separate rabbits were examined. At least three unnotched and notched samples for each property measured were examined in both the longitudinal and transverse directions, specifically in tension both *ex situ* and *in situ* inside the synchrotron X-ray source with real-time simultaneous SAXS measurements. For some conditions, up to eight experiments were conducted.

### Uniaxial tensile tests

Using surgical blades, skin samples with dimensions of 20 × 4 × 0.6 mm^3^ were cut along directions parallel and perpendicular to the backbone of the rabbit. Up to eight samples were tested in each orientation. Uniaxial tensile tests were carried out on an Instron 3342 mechanical testing machine (Instron Corp.) with a load cell of 500 N using the span of 12 mm at strain rates of 10^−1^ and 10^−3^ s^−1^. To keep the specimens hydrated during tests, phosphate-buffered saline solution was sprayed on to the skin samples periodically. The effects of dehydration are explored in Supplementary Fig. 3.

### SEM sample preparation

Strips of the rabbit skin were cut using surgical blade and a steel ruler (the latter to keep the cuts straight). The strips were first immersed in 2.5% glutaraldehyde for 3 h to fix the structure, and dehydrated with an ascending ethanol series (30, 50, 70, 90, 95 and 100 vol.% twice) while preventing shrinkage due to dehydration. The strips were fractured using forceps immediately after being immersed in liquid nitrogen. The fractured samples were immersed in ethanol and dried in a critical point dryer (Auto Samdri 815A, Tousimis). The dried fracture surfaces were then sputter coated with iridium using an Emitech K575X sputter coater (Quorum Technologies Ltd.) and examined by FEI SFEG ultra-high resolution SEM (FEI, Hillsboro).

Samples were also observed under wet conditions using an *in situ* SEM (Hitachi S-4300SE/N SEM (Hitachi America) during the tension testing. However, owing to the wet condition of the skin sample, high resolution could not be obtained. Some stretched samples in different tensile stages (toe, heel, linear and fracture) were prepared using the similar SEM sample preparation procedure (structure fixing, dehydration and critical point drying) and observed using FEI SFEG ultra-high-resolution SEM. All structure-fixed samples, which were tensile tested, were subsequently characterized in the SEM.

### TEM sample preparation

For TEM observation, the skin was cut using a scalpel into 5 mm thick strips. A primary fixation was performed by immersing the tissue sections in 2.5% paraformaldehyde, 2.5% glutaraldehyde in 0.1 M cacodylate buffer for 2 h, and post-fixation was done in 1% osmium tetroxide in 0.15 M cacodylate buffer for 12 h. The specimens were then stained in 1% uranyl acetate for 12 h and dehydrated with an ascending ethanol series, followed by a 1:1 ratio of 100% ethanol and 100% acetone, and finally 100% acetone. Samples were then embedded in Spurr’s low-viscosity resin and polymerized at 48 °C for 48 h. Samples were subsequently sectioned parallel to the skin surface, generating usable samples 70- to 100-nm thick using a Leica Ultracut UCT ultramicrotome (Leica) and a Diatome diamond knife (Diatome). Ultramicrotomed sections were then placed on copper grids for TEM observation, and post stained with Sato lead for 1 min. The glutaraldehyde, which was used to prepare SEM and TEM samples, is a cross-linking agent to fix the structure, which can alter the original orientation of the collagen fibrils; however, the altered angle is within the standard error in this work and did not affect the mechanisms involved.

### Steel model tensile tests

Steels with a circular section (2.38 mm diameter) were used to model the tensile behaviour of a single collagen fibril. The steel wires were shaped into circular segments with radius of 120 mm; the maximum strain capability was prescribed by using different angles of the segments (as shown in Fig. 3b): *θ*=30°, 50°, 70°, 90°, 110° and 130°. The macroscopic strain rate (crosshead velocity divided by total specimen length) was 10^−3^ s^−1^.

### Calculation method

Castigliano’s theorem was used in the derivation for the straightening of an initially circular segment of a steel wire under tension. The strain can be obtained as a function of the change in radius *r*, by simultaneously solving equations 1 and 2.

### Small-angle X-ray scattering

Skin samples with dimensions of 20 × 4 × 0.6 mm^3^ were prepared using surgical blade and sprayed by phosphate-buffered saline solution before testing. A minimum of six hydrated samples were loaded in uniaxial tension at 25 °C at a displacement rate of 40 μm s^−1^ with a span of 4 mm, and exposed to X-rays at beamline 7.3.3 at the Advanced Light Source synchrotron at the Lawrence Berkeley National Laboratory. The mechanical tests were performed with a custom-made rig using a 10-mm displacement stage and an Omega LC703-10 load cell, calibrated to 45 N; this setup permits SAXS data collection to be recorded in real time with the simultaneous measurement of the load-displacement curve. The samples were sprayed by phosphate-buffered saline just before testing, and the entire tensile procedure of one sample took ~4 min.

A Pilatus 1 M detector (Dectris Ltd.), used to collect the SAXS data, was located at the largest allowable distance from the sample (~4 m) to permit detection of the fine changes in the collagen peak positions. The sample was exposed to 10 keV X-rays for 0.5 s at ~5 s intervals during mechanical testing. The SAXS data reduction software Nika was used to calibrate the sample-to-detector distance and beam centre from an X-ray exposure of a silver behenate standard sample. Following this, software written in Labview was used to transform all exposures to polar coordinates (maps of azimuthal angle versus *q*). For the analysis, the first-order peak of intensity versus *q* (=2π/*d*) was analysed, where *d* is the spacing of the peak being diffracted.

Diffuse scattering near the beam centre was removed by fitting a weighted spline function to the scattering curve at each azimuthal angle (the area containing the first-order collagen peak being weighted lightly, and the remainder of the curve weighted heavily), and subtracting this fit from the curve. Azimuthal peaks were then detected and fitted with Gaussian functions to locate the angle of orientation of the collagen. Herman’s orientation factor, also known as *P*2, the second Legendre polynomial, and equal to ½(3cos^2^Φ−1), where Φ is the angle between the orienting entity and fibre axis, was used to quantify the degree to which the azimuthal signal was oriented. Scattering curves were made by integrating the data ±5° from the angle of orientation. The first-order peak of collagen, which had already had background scattering subtracted in a previous step, was then fitted to an exponentially modified Gaussian function, from which peak location, height, area and FWHM were measured. This procedure is given by the sequence of six steps (which are given in Supplementary Fig. 4a–f): (i) image is obtained from Pilatus X-ray detector. (ii) The image is remapped from Cartesian plot to polar coordinates. The background intensity is subtracted with a weighted spline fit. The area between yellow cursors is integrated to create plot of integrated intensity versus angle shown in Step iii. (iii) Peaks are fitted with Gaussian functions to find the central angles of orientation (marked by red cursors). Herman’s orientation parameter, that is, the second Legendre polynomial coefficient P2, is calculated to quantify the degree of orientation. (iv) Areas between yellow cursors (±5° around orientation central angles found in previous step) are integrated to yield two curves of intensity versus *q*, where *q* is defined (in units of Å^−1^) as 2π/*d*, where *d* here is 67 nm, the spacing of the peak being diffracted. (v) The two scattering curves of intensity versus *q* are added to create one curve. (vi) The final curve is fitted with an exponentially modified Gaussian and measurements are made of peak location, height, integrated area and ‘FWHM’.

## Author contributions

M.A.M. and R.O.R. proposed the project and supervised all the work. W.Y., V.R.S. and B.G. performed the experimental testing and characterization; W.Y. and B.G. conducted the SAXS experiments aided by E.S. and P.S. V.R.S. and M.A.M., with W.Y., developed the model. The paper was written by W.Y., V.R.S., B.G., E.S., M.A.M. and R.O.R.

## Additional information

**How to cite this article:** Yang, W. *et al*. On the tear resistance of skin. *Nat. Commun.* 6:6649 doi: 10.1038/ncomms7649 (2015).

## Supplementary Material

Supplementary Information Supplementary Figures 1-4, Supplementary Discussion and Supplementary References
