# Supplementary material for: On the tear resistance of skin
Source: Nat Commun. 2015 Mar 27;6:6649. doi: 10.1038/ncomms7649 (PMC4389263; doi:10.1038/ncomms7649)
Supplement: Supplementary Information — Supplementary Figures 1-4, Supplementary Discussion and Supplementary References [file ncomms7649-s1.pdf]

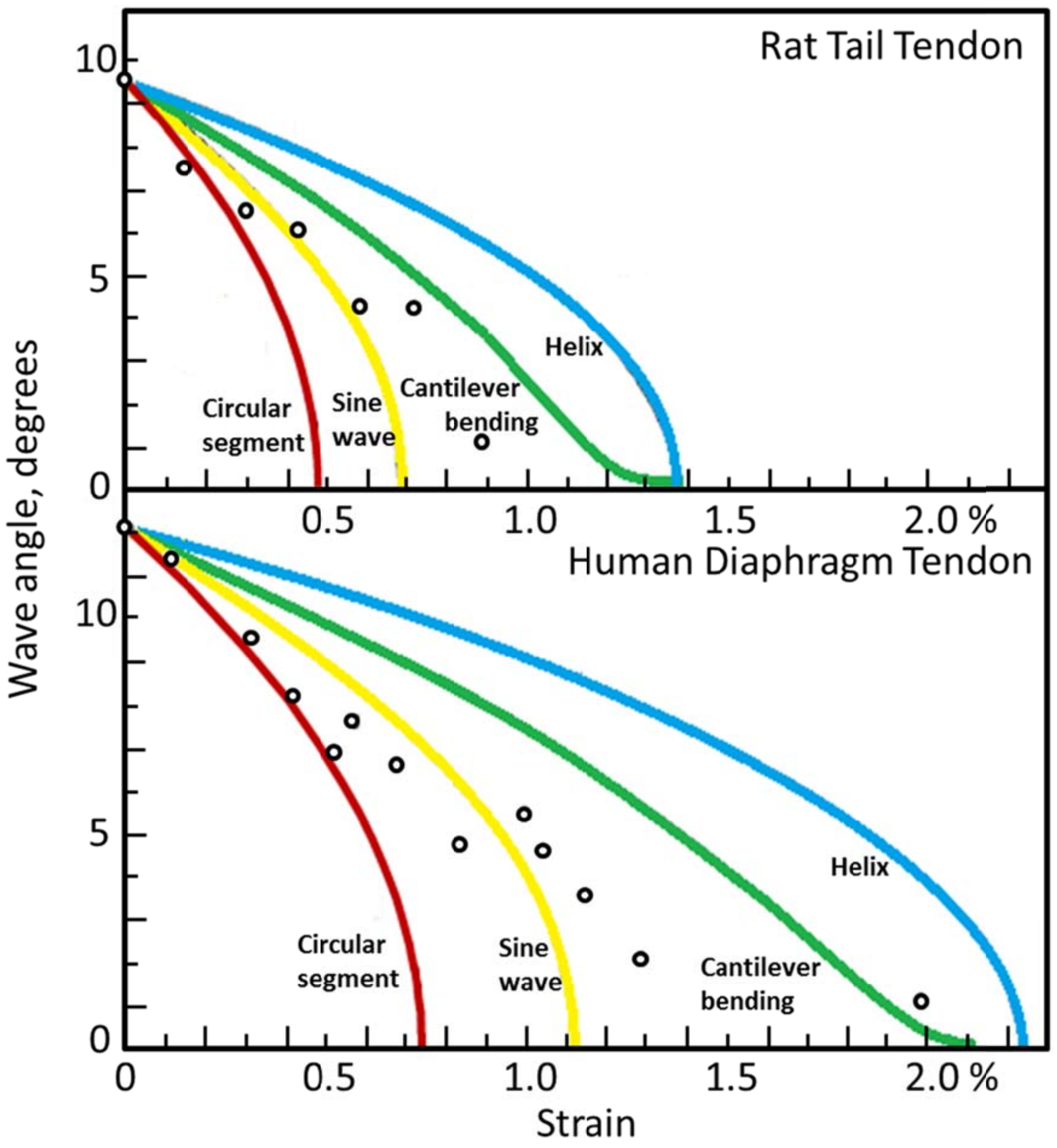

Supplementary Figure 1. Wave angle of tendons under extension. Initial wave angle is 9.6° for rat tail tendon and 12° for human diaphragm tendon. Experimental data points (open circles) show measured wave angle of tendon under extension. Four models are applied for comparison to experimental data; models predict decrease in wave angle as a function of applied strain. The current model developed here uses circular segments. With the incorporation of a viscous term the curve is shifted to the right. The cantilever and helical models over-predict the strain at fixed initial angles of 9.5 (rat tail tendon) and 12° (human diaphragm tendon). Adapted from Dale *et al.*[S2]

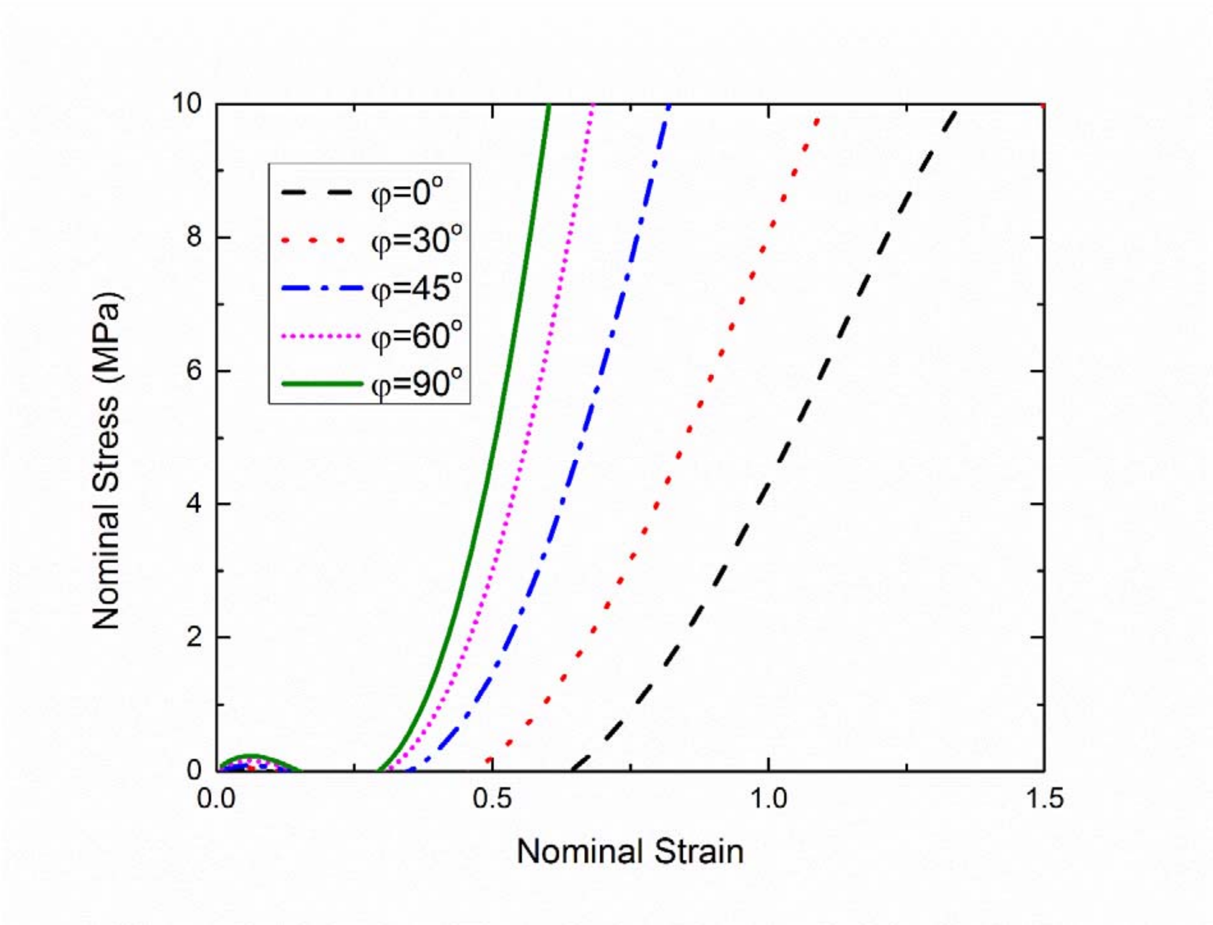

Supplementary Figure 2. Calculated stress-strain curves incorporating anisotropy. Langer line orientation at $\varphi = 90^0$; perpendicular orientation at $\varphi = 0^0$. Orientations of 30°, 45° and 60° have correspondingly intermediate responses. Calculations used Eq. 5 and best fit to experimental data for φ = 0° and 90°.

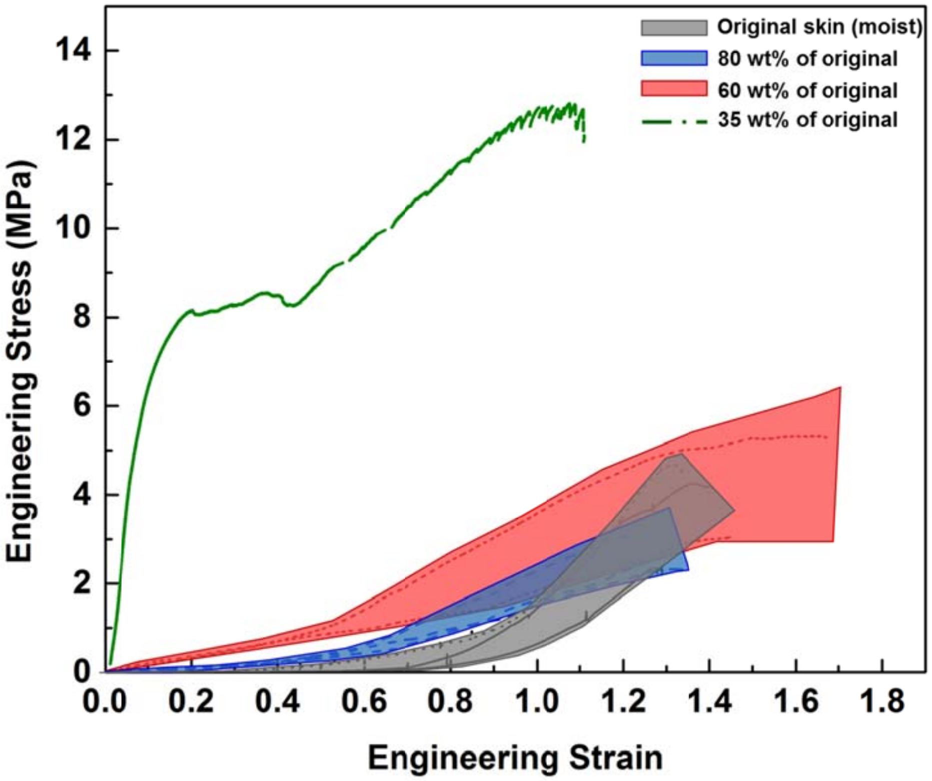

Supplementary Figure 3. Stress-strain scatterband curves of original moist skin and different dehydrated skin (80 wt.%, 60 wt.% and 35 wt.% of moist skin). As skin becomes dehydrated, the toe region of the stress-strain curves becomes shorter and stiffer. After losing 65% weight due to dehydration, the toe region of the stress-strain curve has completely vanished. This is attributed to the loss of sliding between fibers.

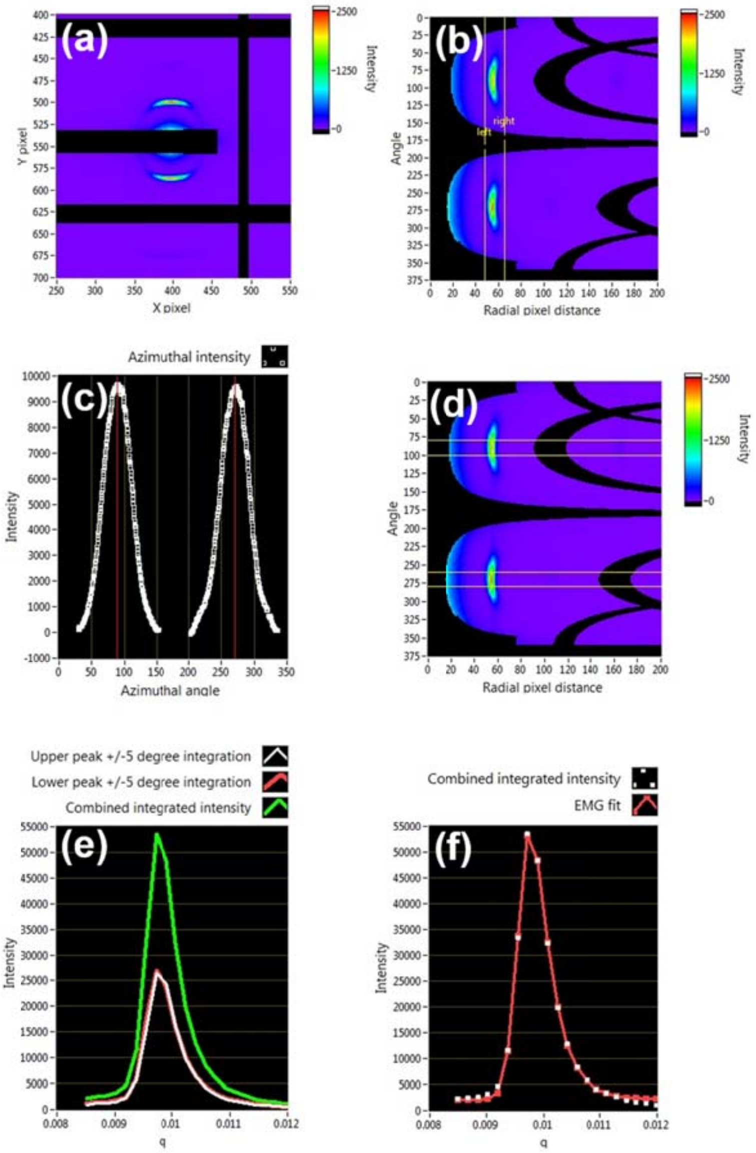

Supplementary Figure 4. SAXS analyzing process with pictures. a) Image is obtained from Plilatus x-ray detector. b) The image is remapped from Cartesian plot to polar coordinates. The background intensity is subtracted. The area between yellow cursors is integrated to create plot of integrated intensity *vs*. angle shown in Step 3. c) Peaks are fitted with Gaussian functions to find the central angles of orientation (marked by red cursors). Herman's orientation parameter $P2$ is calculated to quantify the degree of orientation. d) Areas between yellow cursors (±5° around orientation central angles found in previous step) are integrated to yield two curves of intensity *vs*. $q = 2\pi/d$, where d is the spacing of the peak being diffracted (in our case, 67 nm). e) The two scattering curves of intensity *vs.* q (units of $Å^{-1}$) are added to create one curve. f) The final curve is fitted with an exponentially-modified Gaussian and measurements are made of peak location, height, integrated area and full-width-at-half-maximum (FWHM).

Supplementary Discussion

Selection of the circular segment model

A circular segment model was developed for the deformation of collagen in order to better represent experimental data previously collected. The model builds on the work of Dale *et al.*[1] who recorded the wave angle of tendons under extension, and compared the strain to angle predictions based on the original wave angle and their model. The maximum strain is determined by the angle θ that defines the circular segments, increasing with increasing θ.

For comparison purposes, calculations are shown for a planar sine wave model[2], a cantilever bending (the so-called zig-zag) model[3], and a helical model[4,5] (solid lines). Supplementary Figure 1 is adapted from Dale *et al.*[1] with our circular segment model prediction (red curve) plotted for comparison with experimental data from a rat tail tendon and human diaphragm tendon. Supplementary Figure 1 shows that the cantilever and helical models over-predict the strain at fixed initial angles of 9.5° (rat tail tendon) and 12° (human diaphragm tendon). The circular segment model represents well the experimental data of human diaphragm tendon at a low strains (<0.5%), while the sine wave model fits it better at higher strains (>0.5%). However, the other models do not have a viscoelastic component. The addition of a time-dependent strain shifts the curves to the right, such that the circular segment model could be brought into coincidence with the experimental points. As the simple circular segment model reproduces the stretching of the circular shape of wires, it can be used to explain the stretching of the collagen fibrils at low strains (Stages I, II and the beginning of Stage III). At higher strains, the sliding and delaminating behavior of the collagen can be described by the viscous term.

It is clear that the commonly adopted sine wave model is a more accurate representation of experimental data than the zig-zag or helical models. However, these models all operate under the assumption that there is no sliding between the collagen fibrils. Any amount of sliding will increase the measured strain without affecting the measured wave angle, shifting the experimental points towards the right.

Steel model tensile tests

Predictions of anisotropy using Eq. 5 are shown in Supplementary Figure 2. The responses along and perpendicular to the Langer line orientations are modeled after the ones shown in Fig. 3a. It is clear that the simple model shown in Fig. 4 (Hierarchical level IV) predicts a satisfactory anisotropy. The angle $\varphi = 0^0$ corresponds to the Langer orientation (the stiffest) whereas $\varphi = 90^0$ is the 'softest' orientation. One should, however, be aware that the skin mesostructure is much more complex. For instance, we did not incorporate the presence of elastin nor account for collagen fibers in a non-orthogonal or tridimensional arrangement.

Effect of dehydration on mechanical response

As the skin is dehydrated, its mechanical response is drastically changed. The degree of dehydration was measured by the stress-strain curves for the moist fresh skin; as shown in Supplementary Figure 3, these curves display a long toe region. With dehydration, the toe region becomes progressively shorter and the sample becomes progressively stiffer, with an increase in the slope of the linear portion. Four levels of hydration were tested, corresponding to the percentage of initial weight: 100, 80, 60 and 35%. The first corresponds to fresh skin. The water content of skin is approximately 65-70%. Thus, the skin containing 35% of the original weight has most of the water removed. This was accomplished by drying *in vacuo* for 48 hrs. The stress-strain curve of the most severely dried skin in this work (35 wt.% of the fresh skin which can also be explained as 65% weight reduction from the fresh skin) does not show a toe region at all but displays a high toughness. The sliding between the collagen fibrils is highly limited due to the lack of water molecules. Indeed, the modeling studies of Gautieri *et al.*[6] predict a decrease of the intermolecular separation from 1.6 to 1.1 nm with dehydration and decreased intermolecular sliding in the collagen. In their computations, the corresponding force to pull out a molecule from a microfibril increased from 4 nN to 30 nN. This calculated eight-fold increase is reflected in the measurements presented here. Concomitantly, the change in the shape of the central cut (Figure 1k-l) ceases to take place and a significant part of the tear resistance is lost.

Supplementary References

1. Dale, W. C., Baer, E., Keller, A. & Kohn, R.R. On the ultrastructure of mammalian tendon. *Experientia*. **28**, 1293-1295 (1972).
2 Shadwick, R.E., Russell, A.P. & Lauff, R.F. The structure and mechanical design of rhinoceros dermal armor. *Philos. T. Roy. Soc. B* **337**, 419-428 (1992).
3 Popov, E.P. & Balan, T.A. Engineering Mechanics of Solids. 2nd ed. (Prentice Hall, 1999).
4 Grytz, R. & Meschke, G. Constitutive modeling of crimped collagen fibrils in soft tissues. *J. Mech. Behav. Biomed.* **2**, 522-533 (2009).
5 Comninou, M. & Yannas, I.V. Dependence of stress-strain nonlinearity of connective tissues on the geometry of collagen fibres. *J. Biomechan.* **9**, 427-433 (1976).
6 Gautieri, A., Pate, M.I., Vesentini. S., Redaelli, A. & Buehler, M.J. Hydration and distance dependence of intermolecular shearing between collagen molecules in a model microfibril. *Journal of Biomechanics* , **45,** 2079-2083 (2012).
